# Mr. Laurence, on Vaccination

**Published:** 1806-09

**Authors:** J. Laurence


### *To the Editors of the Medical and Physical Journal.*

GENTLEMEN,

BY publishing my crudities on the origin and specific action of *Vaccina,* in Numb. 2, of your first Vol. I had no expectation of being honoured with the notice of either party; still less, if possible, of furnishing the anti-vaccinists with an auxiliary argument. A spirited writer, however, on that side, Mr. Lipscomb, has incidentally introduced me; and your Reviewer of his publication has facetiously, and I will acknowledge justly enough, remarked on the obscurity and want of precision in certain of my ideas and expressions. But need I, with no higher pretensions than those of mere common sense, be ashamed of having written vaguely on a subject which has hitherto baffled the united talents and physiological researches of the most eminent medical men of our time?

I repeat, and with additional sensibility, my former protestations of the highest respect for the professional character and exalted public services of Dr. Jenner. I think no mortal before him ever had it in his power to bestow so grand and extensive a blessing upon the human race, as the Jennerian discovery will probably be. For, however wary and scrupulous on the subject of modern discoveries, I can, without hesitation, allow to Dr. Jenner the palm of original discovery, by long, accurate, and sedulous experimental exertions, of the reality of the vaccine preventive, which, before, was only an old wife's story, told in two or three obscure corners of the country, unknown and unthought of every where else. I also sincerely hope that this great and patriotic medical character will soon receive an additional remuneration, more adequate to his long continued labours, the immensity of the public benefits resulting from them, and more suitable to the high liberality of this country. Indeed, Dr. Jenner has a just claim on the gratitude of every country, and every government in the world.

But searchers after truth must not be dazzled with the lustre of great names, nor take for granted the infallibility of any man, or any system. And I do not feel disposed to attach any great consequence to the hypothesis, that we have originally derived our diseases from brute animals, or to make use of it as a ground for the notion, of the pox of the cow proceeding from the grease of the horse's heel.

Diseases

Diseases are doubtless intercommunicable between men and beast; most of the morbid seeds will thrive, perhaps, reciprocally in either soil, as will those of animal generation. Still nature may preserve a more regular course than appears to our limited powers of research.

I will freely own, that the point in dispute would be at once settled and determined for ever, could we depend on the accuracy of those accounts from the continent, where it seems they are in the constant habit of inoculating indifferently from equine or vaccine matter, and with similar successful results. But I am afraid our experience will by no means authorize us to encourage such dependence. We have received strange and incongruous accounts from abroad. The mange and scab in sheep have been confounded with the pox; and those animals have, we are informed, been vaccinated with the expectation of preventing the former. It has been pretty generally acknowledged, both on the continent and in this country, that the grease in horses is not understood; a very ill complement, surely, to so many scientific and practical veterinary writers, who have amply described that malady in the horse, so extremely common, and so extremely obvious in its nature, causes, and effects.

To proceed to the arguments in favour of the above hypothesis in England:—I apprehend it will obtain little additional credit, in consequence of the late intelligence communicated by the Doctors Jenner and Adams, from Mr. Tanner's farm, near Berkeley (Medical Journal, No. 88.) Those philosophers, laudably in search after facts, doubtless offered such as they obtained for the purpose of discussion, that such might thence have their just value, but not more allowed them. The proof in this case is merely presumptive. The boy might carry a sufficient quantity of the greasy matter for the purpose of infection, upon his hands, to the teats of the two cows, or he might not; having, according to laudable and cleanly usage, wiped the said matter clean from his fingers upon, and therewith vaccinated, or equinated, his coat sleeves, or his sheep-skin breeches. In the mean while the two cows first infected, might have received the infection from a different source, or have spontaneously produced the disease; and from those the infection might, or might not, have proceeded to the rest of the herd. It farther very well might not have been even so, since the herd might have taken the disease in succession, one after the other, and all from the same source, probably contagion of the

 atmosphere; atmosphere; and perhaps the state of the air, from the long prevalence of N. E. winds might be a predisposing cause of the diseases. As to our actual experiments in this country, some experimenters have produced, from horse grease, *a disease similar to cow-pox*. Dr. Head (Lond. Med. Soc.) *thinks*, that, in one case, he had evidence that cow-pox was derived from the grease. But we have a number, on the other hand, who never could, after repeated fair trials, produce either pox or any other disease from the insertion of the *virus* of grease from the horse into the teats of the cow; among these Dr. Kinglake has published in your 88th No. several very satisfactory experiments. One of the arguments made use of in favour of this hypothesis, appears to me, if not fatal to it, yet demonstrative of its very uncertain nature. It has been often urged as a plea for want of success, that the proper species of grease has not been obtained. With respect to that general train of arguments which might be brought to bear powerfully against the presumed virtue in equine humours, they will suggest themselves readily to every one who has reflected on the subject; but, instead of arguments, I follow Dr. Kinglake, in calling for experimental facts, than which nothing can be more easily attainable, since the fluid of grease may be, *miserable dictu*, always found near the head quarters of *vaccina*, of all species and qualities, and in every possible stage. Surely, as the Dr. says, sufficient experiments of this kind might be instituted with the cow; and with all manner of safety, to my thorough conviction, with the human subject, provided the matter were taken from the grease in its acute or first stage; and if I understand the matter aright, we make use of the variolous, or vaccine humour in its mature, not in its putrid and acrid state; analogically, we therefore ought to do the same with the fluid of grease. But, *perhaps*, whilst I am disputing the point, the irrefragable evidence of facts has already sealed my refutal, to which I shall then bow with submission, without being in the smallest degree ashamed of the ground on which I now stand.

If we must account for cow-pox with more solicitude than we are accustomed to do for other diseases, for my own part, as to its *first* ætiology, I guess it was originally by nature bound up in the volume of the cows constitution. In the same light I view the greasy humour (to use the language of the stable) so peculiarly abundant in the body of the horse, and so apt to extravasate and overflow, in in various circumstances and through diverse channels. The cow-pock appears to me, a mighty easy thing to say, to be the *variola* of that genus of animals. I think the same of the sheep and swine-pox. That there should not exist a precise analogy between these and the human disease of similar denomination, I apprehend to be of no material consequence to the argument. Nature has not thought proper to entrust us with all her secrets, and we may perhaps never arrive at the knowledge of the necessity, by which she was compelled to make *variola* a recurrent in brute, and a non-recurrent disease in human creatures; or why, considering that anomaly of rule, the one should supersede the other.

But proofs of the sufficiency, safety, mildness, and pleasantness of vaccination, as a preventive of the small-pox, have been, through a course of years, so multitudinous and universal over the whole civilized earth, that all theoretic discussions of the why and the wherefore may be safely suspended, and left to the gradual and fortuitous elucidation of time; whilst the immediate object ought to be, to catch at the proffered benefit of such ineffable consequences to the human race; and they must have miserable souls, truly, who, with the mountains of evidence before their eyes, of absolute security, on such easy terms, against a horrid and loathsome pest, are yet afraid to venture. Never before, was any discovery pursued with such rapid steps, and upon so grand and universal a scale. Nor is the noble enthusiasm of individuals and public bodies yet spent, or at all slackened, after all their great exertions; and our present patriotic administration, the decided and fatal enemies of that first and most crying of all human abominations, the Slave Trade, have given notice, that they are about to take *Vaccina*, the friend of human nature, under their especial protection. The object of Lord Henry Petty appears to be, to devise proper means for enlightening the minds, and counteracting the groundless prejudices of the people, on this subject, so important to their welfare; to the end, that as early as possible the advantages of vaccination may be rendered universal, and the plague of small-pox be totally stayed and finally extinguished.

I am uncertain whether it occurred to my own mind, or whether I somewhere read of a plan, which seems to me to be one of the most feasible which can be conceived to spread universally the practice of cow-pock inoculation. It is, *to commit the task of admonition to the hands of the ministers*

*ters of all religions.* When they baptize or name a child, they might at the same time, as a regular branch of their duty, set forth to the parents, in their most forcible and persuasive mode, the great advantages, public and private, and the strong necessity of infant inoculation. With this advice, a short printed account of the facts which demonstrate the success and safety of the cow-pox should be given gratis. Such printed accounts should be, from time to time, circulated throughout every parish, and most particularly amongst the lower classes, at the expence of government. Man is the creature of custom, good or bad. The inducing a habit is every thing; and could it once become as necessary, that is to say, as customary to vaccinate as to name a child, the small-pox would not now have existed. Indeed, the extinction of that foul and fatal disease has ever been in the power of the people themselves, since the discovery of inoculation by similar means; but it was formerly at a considerable risk of life, which by the vaccine discovery is reduced even to nothing.

Having suffered much myself from a waste of bodily strength, and otherwise, under the natural small-pox, against which disgust so penetrated my infant mind, that a lapse of full fifty years has not been able to eradicate the vestiges of it, I determined to inoculate my children at the breast; one of whom, a boy, had nearly perished under inoculated small-pox, but he survived to be buried, seventeen years afterwards, in the Indian Ocean. In May 1803, I caused to be vaccinated, at St. Pancras Hospital, my female infant, of the age of six months. She was then teething, with a rash upon her, and considerably affected by influenza. The inoculation did not succeed, which I attributed to a medicine taken from the hospital in too powerful a dose, the same quantity having occasioned super-purgation in a neighbour's child of a robust habit. After my child's recovery, I caused her to be again vaccinated, and she went through every stage of the disease regularly and most successfully, the pustules being very beautiful, and assuredly genuine. Accidently or not, her state of health mended with the progress of the cow-pox.

In the following July, the child had an eruption resembling the tooth rash, without itching or inflammation; afterwards a small pimply eruption, which itched violently. In the autumn, a pustulous eruption spread thickly over her lower extremities, appearing, I believe, no where above, excepting upon her mouth. Many persons who saw saw it said, 'it was the cow-pox come again.' The pustules were distinct, having the areola around them, scabbing off regularly, and leaving a blue spot upon the skin. They strongly resembled pustules which I have seen on the teats of cows; and probably this is the eruption which the old women call 'the scurvy after the cow-pox.' The same humour recurred the following October, 1804; again, July, 1805; and a remaining pustule was visible on one foot in November. The quantity of eruption diminished every return, and has totally ceased this year. No external applications had any effect in counteracting or dispersing this eruption, any otherwise, than if repelled or dispersed in one part, it re-appeared in another. A few mild doses of calomel seemed to accelerate its dispersion. The child is of a delicate habit, but of a constitution perfectly sound and healthy; and her state of health, according to my usual custom, has been superintended from the hour of her birth with the utmost prophylactic care in my power to bestow. She was in perfect health and spirits previously, during the continuance and subsequently to the cessation of each eruption; and granting it to bear any possible relation to the cow pox, of which I am an incompetent judge, I can estimate it as no objection, probably the reverse. This child has lately had the chicken-pock, but never the hooping-cough or measles; both which, a certain fancy I have long had in my head, leads me to suppose may be prevented.

I ought, however, to observe, that when the eruption in question first appeared on the infant, my other children caught from the children of a chairwomen, accidentally employed, a humour which, I found on enquiry, was supposed to have been brought into the woman's family by some nurse-children. According to the woman's account, this eruption first appeared in "*broad, white, bladdery bumps.*" When I examined these children, their fingers were distended with pustules; their hands, sides, and hams covered with them; and the disease appeared to me to wear the direct psoric character, and to be inveterate pustulous itch. The pustules, on their decline, left indurations on the hands, which were to be felt many months afterwards. One of these children had suffered severely, the previous year, from the inoculated small-pox, of which she now bears the evident marks. This eruptive disease, however, was said to be almost epidemic in the neighbourhood during the following autumn, many distinct and unconnected families being similarly affected by it; and it was was declared by several of the apothecaries attending, not to be the itch, but an eruptive affection connected with the peculiar state of the atmosphere. The most powerful unguents and lotions which I could employ, amongst the former, that recommended by Mr. Ring, had no certain or visible effects; the case was similar with internal remedies; whence I was induced intirely to desist, and, in a few months, the eruption disappeared spontaneously, and most probably from a change of temperature in the weather. The character and appearance of the eruption upon the infant still seemed distinct from that of the other children; there might yet be an affinity between the two, which I did not possess the skill to discover, and I omitted to state, that about two months previously to the inoculation of the infant, there appeared on the soles of her feet, arms, and thighs, a thinly scattered eruption of firm, dry pustules, which soon died away, without any perceptible consequences.

The opposition to the cow-pock inoculation was quite in the course of human affairs, and ought not to have given the least surprize or, indeed, regret to the experienced. There must ever exist various motives for opposing new improvements. We have a sect of alarmists, who, notwithstanding so immense a portion of the habitable globe remains yet unpeopled and uncultivated, exist yet under the direful apprehension of perishing at last with famine, from the present rapid increase of population, which they loudly call upon legislators to check; not indeed absolutely by converting the surplus into Abelards, but by a not altogether dissimilar process. Others there are, who dread a decrease of the consoling resources of pestilence and war, and are even jealous of the diminution of vice, crime, and misery, lest human profit should diminish in proportion. Such sentiments are no secrets. But I have ever conceived, that the opposition publicly and fairly made to vaccination has rendered the highest degree of service to the practice, by placing it upon its proper and legitimate basis of truth, far earlier than could otherwise have happened; and have always regretted that the respectable Mr. Goldson was treated with such an undeserved harshness. Right of opinion, in all possible cases, is universally reciprocal, and naturally inherent. It is a precious property, which, instead of being taken from a man by the law, by the public, or by individuals, ought to be the first thing unconditionally secured to him. And what right to his opinion concerning *vaccina* had any man which which Mr. Goldson did not also possess? As to the bull-faced, chimerical, and bloody-boned opposition, every man who runs may read the intent and drift of it. We are by no means bound to suppose the authors themselves believed all they published as truths, since we know of old that truths are of two sorts, the actual and the virtual. One of the sages of this class, about two years since, farther obliged the lovers of the ridiculous and the wonderful, by publishing a most ostentatious account, how a serpent jumped down his dog's throat! and how, for humanity's sake, he kept the miserable and tortured animal alive during four and twenty hours. The system of terror, however, had its desired effect upon many of those to whom it was addressed, namely, the ignorant and vulgar; and the consequence was a temporary spread of the contagion of the small-pox, by which some hundreds were relieved from all farther anxiety about the respective merits of the two modes of inoculation; and this last struggle of opposition has effectually served the cause it was intended to injure. Men are seldom informed or reformed, without the chastisement of suffering; and in this case, the living will profit by the example of the dead.

I must beg leave, in few words, to notice an inaccuracy in my former communication, (Vol. I.) where I identified the *heavings* with the pox in swine. The heaving, indeed, is frequently a symptom of that disease, but it is a general term for an inflammation of the lungs. With sincere acknowledgments for the instruction and entertainment I am constantly reaping from your valuable Journal.

I am, &c.

J. LAURENCE.

